# Case Report: Early diagnosis of LAD-III in newborn with persistent leukocytosis and hemangioma-like lesion of the urinary bladder

**DOI:** 10.3389/fped.2025.1550643

**Published:** 2025-03-07

**Authors:** Riccardo Pagani, Laura Lorioli, Francesca Favini, Eleonora Severi, Marco Salvi, Lidia Pezzani, Maria Iascone, Lucia Migliazza, Claudia Pellegrinelli, Maurizio Cheli, Massimo Provenzi, Giovanna Mangili

**Affiliations:** ^1^ Department of Pediatrics, Milano-Bicocca University, Fondazione IRCCS Ospedale San Gerardo dei Tintori, Monza, Italy; ^2^ Department of Neonatology, ASST Ospedale Papa Giovanni XXIII, Bergamo, Italy; ^3^ Department of Pediatrics, ASST Ospedale Papa Giovanni XXIII, Bergamo, Italy; ^4^ Laboratory of Medical Genetics, ASST Ospedale Papa Giovanni XXIII, Bergamo, Italy; ^5^ Department of Pediatric Surgery, ASST Ospedale Papa Giovanni XXIII, Bergamo, Italy

**Keywords:** primary immunodeficiency, leukocyte adhesion defect type III, FERMT3 gene, kindlin-3, bladder hemangioma, newborn

## Abstract

Leukocyte Adhesion Defects (LADs) are a group of rare autosomal recessive immune disorders characterized by constitutional defects in the process of leukocyte migration. Among these, LAD-III is the rarest, with only a few cases documented in scientific literature. It is caused by mutations in the FERMT3 gene, impairing integrin function in both white blood cells and platelets. Thus, patients exhibit a variable degree of immunodeficiency along with a severe bleeding tendency referred to as “Glanzmann-like”, due to dysfunctional platelet GPIIb/IIIa. The diagnosis of LAD-III is typically made in infancy or early childhood, following medical evaluations for recurrent infections and bleeding episodes. Here we report the case of a female newborn admitted to our NICU at day four of life with a history of petechial rash and gross hematuria. Radiological and endoscopic assessments revealed a hemangioma-like lesion of the bladder wall. Blood exams showed persistent leukocytosis without signs of infection, associated with mild thrombocytopenia and normocytic anemia. Notably, platelet function assays demonstrated defective aggregation with all agonists tested. Next generation sequencing analysis identified a homozygous nonsense mutation in the FERMT3 gene, ensuring early access to hematopoietic stem cell transplantation, which is the only curative treatment. To the best of our knowledge, this is the first reported case of LAD-III diagnosed in the neonatal period and the first to associate this rare disorder with bladder angiomatosis. This case highlights the importance of early genetic evaluations in newborns with unexplained hematological abnormalities and bleeding tendencies.

## Introduction

Leukocyte adhesion is a critical process in the immune response, facilitating the migration of white blood cells to sites of infection or injury. This process involves several sequential steps and specific adhesion molecules expressed on the surface of both leukocytes and endothelial cells. The rolling phase involves loose adhesion of leukocytes to the vessel wall, mediated by the interaction between selectins on activated endothelial cells and their sialylated ligands, which are constitutively expressed on leukocytes. Rolling leukocytes are activated by chemokines released from the inflamed tissue, which induce conformational changes in integrins through a complex G-protein signaling cascade (so called integrin inside-out activation). Activated integrins have a higher affinity to their ligands on endothelial cells, leading to firm adhesion and subsequent diapedesis of leukocytes (1). Constitutional defects in this process define a group of rare autosomal recessive immune disorders known as Leukocyte Adhesion Defects (LAD) characterized by delayed umbilical cord separation and recurrent bacterial and fungal infections often manifesting in infancy. While LAD-I and LAD-II are caused by mutations that affect the expression of integrins and selectin ligands, respectively, LAD-III (previously referred to as LAD-I/variant) is characterized by mutations in the FERMT3 gene encoding for kindlin-3, which is fundamental for the inside-out activation of integrins (1, 2). Despite normal surface expression of integrins on granulocytes, lymphocytes and platelets, cells fail to activate in response to stimuli. Therefore, patients experience a mild immunodeficiency along with a severe bleeding tendency akin to Glanzmann thrombasthenia (GT) due to dysfunctional platelet GPIIb/IIIa (integrin αIIbβ3). To the best of our knowledge, in this report we describe for the first time in scientific literature a case of LAD III in a newborn presenting with persistent leukocytosis, transient hematuria and a hemangioma-like lesion of the urinary bladder wall.

## Case description

**Figure 1:**
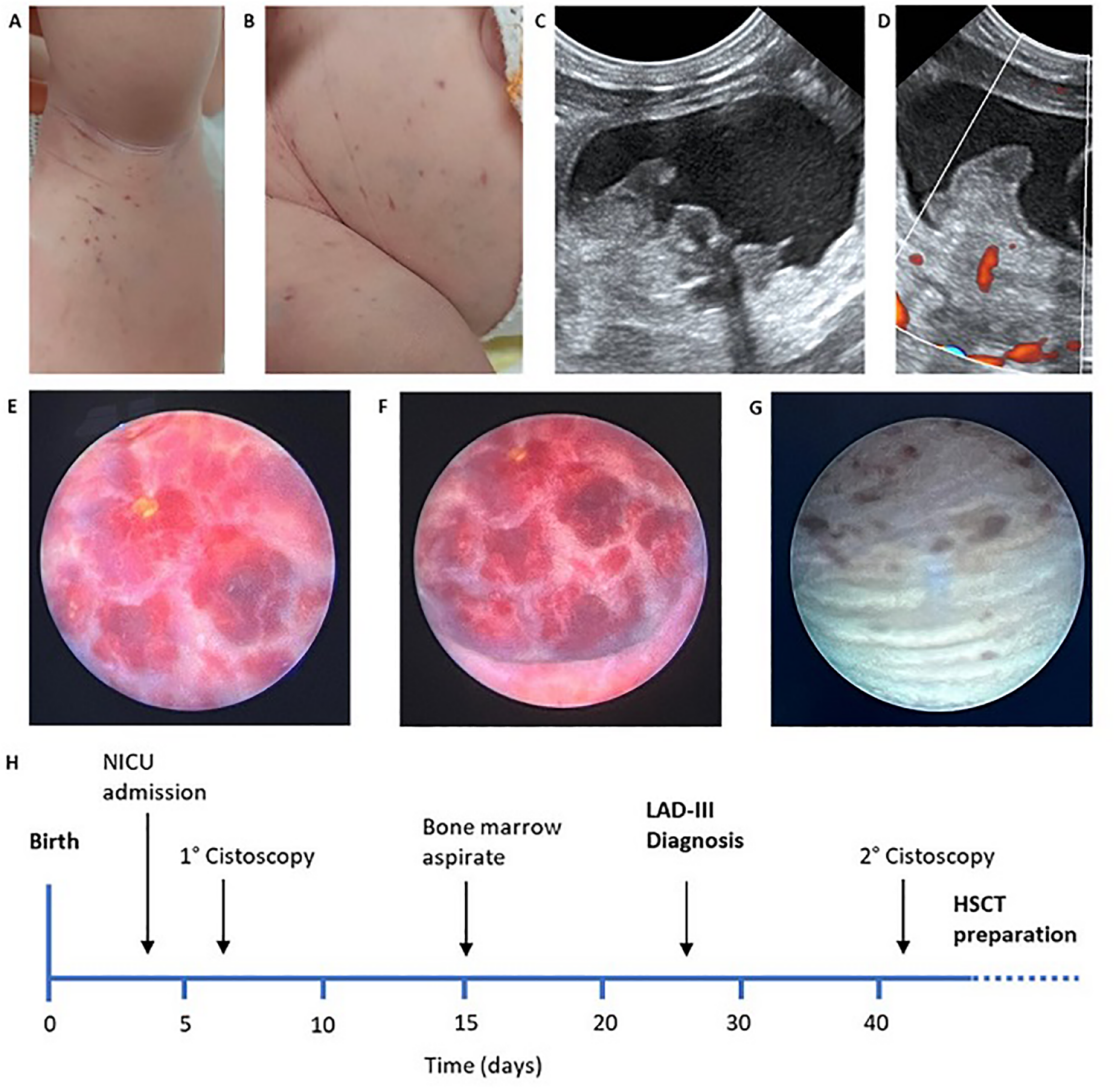
**(A,B)** Petechiae and ecchymoses over the trunk and limbs at admission; **(C,D)** early abdominal ultrasound showing vascularized endoluminal bladder wall mass; **(E,F)** early cystoscopy showing well demarcated hemangioma-like lesion of the bladder wall; **(G)** control cystoscopy after treatment showing almost complete resolution; **(H)** clinical history timeline.

**Figure 2:**
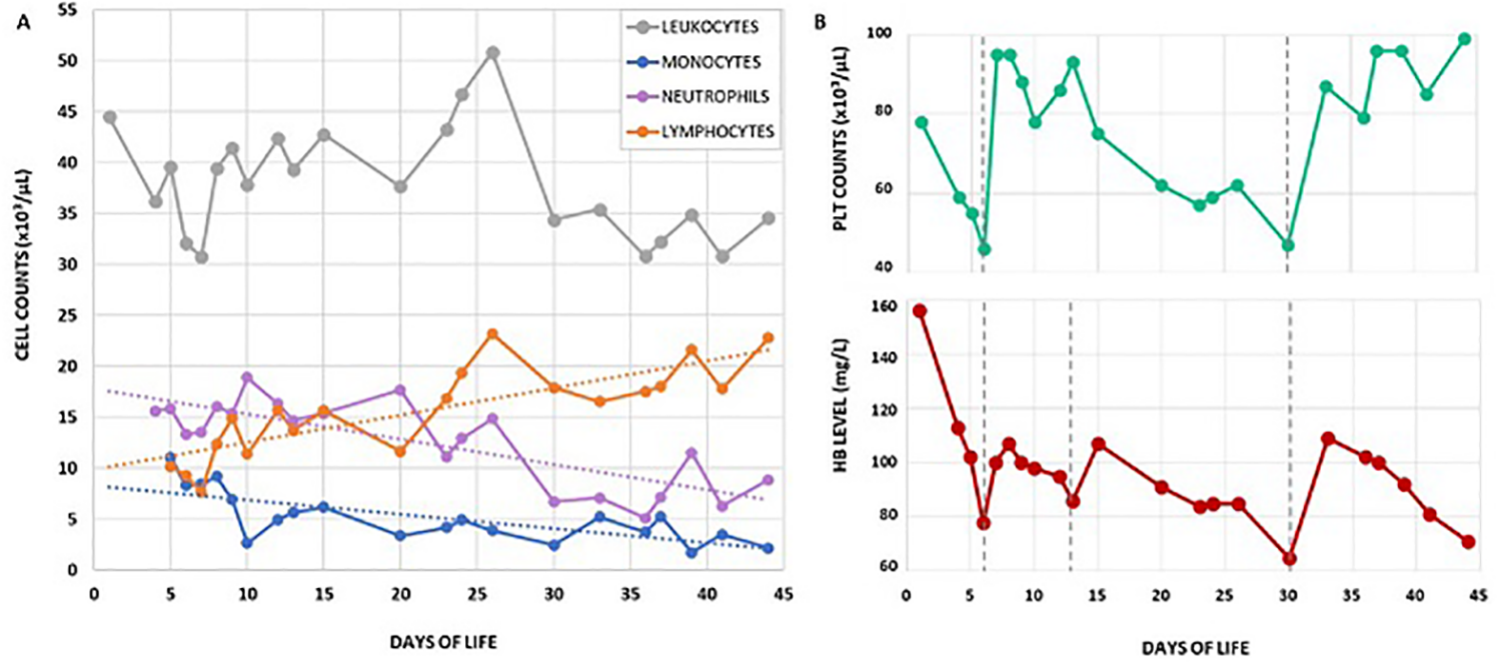
**(A)** Leukocyte differential counts over the first 43 days of life. Neutrophils and monocytes were the dominant subsets during the first weeks of life, while lymphocytes gradually increased over time; **(B)** hemoglobin level and platelet counts over the same period of time. Grey vertical dotted lines indicate red blood cell and/or platelet transfusions.

We present the case of a female neonate, second child of a consanguineous couple of Turkish origin (first cousins), born at 39 weeks of gestational age by vaginal delivery, weighing 3,260 g. The pregnancy was uneventful except for insulin-treated gestational diabetes; maternal TORCH serology was negative and there was no relevant family history. The baby was admitted to our NICU at day 4 of life with a history of diffuse petechial rash since birth and hematuria starting from the third day of life. Blood counts at birth showed leukocytosis and moderate thrombocytopenia (Hb 15.6 g/dl WBC 44.500/mm^3^ PLT 78.000/mm^3^) with normal coagulation and negative inflammatory markers. At the time of admission, the baby was in good general condition and gaining adequate weight. Multiple petechiae and ecchymoses over the trunk and limbs were present, which resolved over the course of a few days (Figure 1A,B); the umbilical stump showed serous discharge. Gross hematuria was initially addressed with bladder ultrasound, which showed a mass projecting inside the lumen (Figure 1C). This finding was initially interpreted as an organized blood clot. However, the identification of intralesional vascularization on Doppler imaging (Figure 1D), in addition to further assessment through pelvic MRI (data not shown), prompted the decision to perform cystoscopy for a more comprehensive evaluation of the bladder wall. During the procedure, the bladder was extensively irrigated with saline to remove all blood clots, revealing a well-defined, round lesion on the bladder wall. The macroscopic appearance of the lesion was consistent with a diagnosis of bladder hemangioma, as confirmed by the pediatric urologist (Figure 1E,F). Diagnostic biopsy was not performed due to the hemorrhagic risk. 5 weeks oral propranolol treatment (2 mg/kg daily) was started together with local methylprednisolone instillations (10 mg daily) for a total of 10 days. Hematuria resolved within a few days and control cystoscopy showed an almost complete regression of the lesion (Figure 1G). Over the first month of life the proband's blood exams showed progressive normocytic anemia and moderate thrombocytopenia both requiring multiple transfusions, associated with persistent leukocytosis (mainly monocytosis and neutrophilia) without clinical or laboratory evidence of infection or bleeding (Figure 2). In addition, blood smear revealed high counts of myeloid precursors (namely metamyelocytes and myelocytes) and erythroblasts. Over the first month of life monocyte and neutrophil counts progressively decreased, while lymphocyte counts increased. Hematologic malignancy was ruled out by peripheral blood immunophenotyping and bone marrow aspirate analysis. Extended coagulation profile was normal at multiple timepoints. Platelet function tests showed defective aggregation with all agonists including ristocetin (Table 1), while flow cytometry for platelet receptors GPIIb, GPIb and GPIIIa (CD41, CD42b and CD61, respectively) was normal. Thus, we performed Whole Genome Sequencing (WGS) on the baby and her parents, which identified a homozygous nonsense mutation in the FERMT3 gene. Both parents were found to be carriers of the same mutation. The patient was therefore referred to the pediatric immunology center for hematopoietic stem-cell transplantation (HSCT).

**Table 1:** Results of platelet aggregation studies.

Agonist	Proband	Normal control
ADP 2 μM	1%	>80%
ADP 10 µM	2%	>80%
Epinephrine 5 µM	2%	>80%
Collagen 1 µg/ml	0%	>70%
Collagen 4 µg/ml	0%	>80%
Arachidonic acid 0.5 mM	1%	>80%
Ristocetin 1.5 mg/ml	4%	>82%

## Discussion

LAD-III is an extremely rare disease with approximately 60 cases reported in scientific literature from around 52 families, mostly of Middle Eastern origin (3). Patients are usually diagnosed in infancy and early childhood, following medical assessments for recurrent infections, bleeding diathesis and persistent hematological abnormalities. The management of LAD-III involves blood transfusions and prompt broad-spectrum antibiotic therapy in case of infection; definitive treatment is based on early HSCT (4, 5). To the best of our knowledge, there are no clinical reports documenting the diagnosis of LAD-III during the neonatal period. This case report presents a newborn with a history of petechial rash at birth and a hemangioma-like lesion of the urinary bladder causing gross hematuria. Early genetic testing revealed the presence of the homozygous nonsense mutation p.Arg573X (c.1717C > T) in exon 14 of FERMT3 gene (OMIM 607901), which generates a truncated kindlin-3 protein. The variant is classified as likely pathogenetic on the ClinVar database (6) and has been previously reported in literature as causative of LAD-III (7). Kindlins are critical mediators of integrin inside-out signaling: they bind to the C-terminal region of the β-integrin subunit and enhance talin-mediated integrin activation (2). Since kindlin-3 is predominantly expressed in hematopoietic cells, its loss of function disrupts the proper activity of β_1_ and β_2_ integrins on the surface of granulocytes and lymphocytes, and β_3_ integrin (GPIIb/IIIa) on the surface of platelets. Consequently, leukocytes fail to migrate from the bloodstream to inflammatory sites, platelets fail to aggregate in response to injury (8).

In the present case, persistent anemia, leukocytosis and thrombocytopenia with impaired platelet aggregation were the key clinical features that triggered genetic evaluation. Transfusion-dependent anemia is a frequent finding in LAD-III. While many authors suggest it is a consequence of chronic blood loss and iron deficiency, recent works have demonstrated that kindlin-3 defective red blood cells exhibit abnormal shape and function (9). Furthermore, studies on kindlin-3 defective mice showed that integrin inside-out activation is critical for the adhesion of hematopoietic cells in the bone marrow niche and ultimately for normal erythropoiesis (10). As for leukocytosis, our patient showed significant neutrophilia with high counts of circulating myeloid precursors since birth. Over the course of the first month of life, granulocyte and monocyte counts decreased while lymphocytes progressively increased. The differential diagnosis of leukocytosis in the neonatal period includes sepsis, leukemoid reaction and lymphoproliferation [e.g., juvenile myelomonocytic leukemia (JMML), infant acute leukemias and transient abnormal myelopoiesis of Down syndrome] (11). While infection was ruled out by monitoring inflammation markers and cultures, malignancy was excluded through peripheral blood and bone marrow cytologic and immunophenotypic analysis. Moderate persistent thrombocytopenia with impaired platelet function was a prominent diagnostic element in our patient. Remarkably, despite laboratory evidence of complete lack of platelet aggregation, cutaneous bleeding resolved within the first days of life and hematuria promptly subsided following appropriate management of the bladder angiomatosis. Since then, no signs of active bleeding have been observed. The differential diagnosis of impaired primary hemostasis in newborns comprises von Willebrand disease, Wiskott-Aldrich syndrome, inherited platelet dysfunction disorders [e.g., GT, Bernard-Soulier syndrome and ADP receptor defect] and vascular anomalies (e.g., Kasabach–Merritt syndrome). Thrombocytopenia has previously been reported in several case reports of LAD-III. Integrins are expressed on bone marrow megakaryocytes and have been described to play an important role in lineage development and cellular functions, such as adhesion to bone marrow stroma and proplatelet formation (12). We therefore suggest that in LAD-III, kindlin-3 deficiency might impair the physiology of megakaryocytes. The platelet dysfunction observed in LAD-III is referred to as “Glanzmann-like”, implying a defective aggregation with all agonists except ristocetin, as shown in most reports. Indeed, ristocetin bypasses GPIIb/IIIa and induces platelet agglutination through the GPIb-IX complex signal. Nevertheless, our proband's platelets failed to aggregate even when exposed to ristocetin. Notably, *in vitro* studies have shown that defective integrin activation in the presence of specific kindlin-3 mutations might also affect GPIb-IX function in platelets (9). It is therefore possible that LAD-III platelet dysfunction and bleeding phenotype may vary depending on the patient-specific FERMT3 mutation.

The association of LAD-III with angiomatosis of the bladder has never been described before in literature. Interestingly, recent *in vitro* studies have shown that kindlin-3 is also expressed in endothelial cells, where it might have a role in regulating angiogenesis (13). Furthermore, suppression of β1 integrin signaling in endothelial cells has been shown to promote infant hemangioma development via constitutive activation of VEGFR2 signaling (14, 15). We therefore speculate that loss of function of kindlin-3 in endothelial cells could impair β1 integrin activation and lead to hemangioma initiation in a similar manner.

In conclusion, the diagnosis of rare immune disorders like LAD-III during the neonatal period is challenging, and requires a close collaboration among pediatric specialists. Newborn patients are complex to approach in clinical practice, as they hardly exhibit the typical signs and symptoms of underlying conditions, and diagnostic tools are often limited or not readily available. Furthermore, primary immunodefiencies in newborns are rarely documented in literature and are often underdiagnosed due to the limited use of genetic testing. We therefore wish to emphasize the importance of early genetic counseling, particularly in cases of consanguinity. Early diagnosis is critical in LAD-III, as timely prevention and management of infections and severe bleeding episodes can greatly enhance patient's prognosis. It also allows for early selection of a highly matched bone marrow donor, which is essential for reducing the incidence of post-HSCT complications and improving the patient's quality of life.

## Data Availability

The original contributions presented in the study are included in the article/Supplementary Material, further inquiries can be directed to the corresponding author.
